# Estimation of Individual Exposure to Erythemal Weighted UVR by Multi-Sensor Measurements and Integral Calculation

**DOI:** 10.3390/s20154068

**Published:** 2020-07-22

**Authors:** Wenwen Cheng, Robert Brown, David Vernez, Daniel Goldberg

**Affiliations:** 1 College of Architecture, University of Oklahoma, Norman, OK 73019, USA; 2 Department of Landscape Architecture and Urban Planning, Texas A&M University, College Station, TX 77843, USA; rbrown@arch.tamu.edu; 3 Center for Primary Care and Public Health (Unisanté), Department of Occupational and Environmental Health, University of Lausanne, CH-1015 Lausanne, Switzerland; David.vernez@unisante.ch; 4 Department of Geography, Texas A&M University, College Station, TX 77843, USA; daniel.goldberg@tamu.edu

**Keywords:** individual UVR exposure, multi-sensor UVR measurement, children UVR health

## Abstract

Ultraviolet radiation (UVR) can be hazardous to humans, especially children, and is associated with sunburn, melanoma, and the risk of skin cancer. Understanding and estimating adults’ and children’s UVR exposure is critical to the design of effective interventions and the production of healthy UVR environments. Currently, there are limitations to the ways computer modeling and field measurements estimate individual UVR exposure in a given landscape. To address these limitations, this study developed an approach of integral calculation using six-directional (up, down, south, north, east, and west) field-measured UVR data and the estimated body exposure ratios (ER) for both children and adults. This approach showed high agreement when compared to a validated approach using ambient UVR and estimated ER data with a high r-square value (90.72% for child and adult models), and a low mean squared error (6.0% for child model and 5.1% for adult model) in an open area. This approach acting as a complementary tool between the climatology level and individual level can be used to estimate individual UVR exposure in a landscape with a complicated shady environment. In addition, measuring daily UVR data from six directions under open sky conditions confirmed that personal dosimeters underestimate actual individual UVR exposure.

## 1. Introduction

Based on wavelength, ultraviolet radiation (UVR) is subdivided into UVA (315–400 nm), UVB (290–315 nm), and UVC (200–290 nm). The UVC band is the most energetic radiation but does not penetrate the atmosphere. UVB and UVA that penetrate the atmosphere have the highest biological impact to human health. 

Specifically, skin cancer is one of the most significant public health problems. More people are diagnosed with skin cancer each year in the U.S. than all other cancers combined [1]. UVR has been associated with three major kinds of skin cancer: basal cell carcinoma, squamous cell carcinoma, and cutaneous malignant melanoma [2]. According to the American Cancer Society, over 100,000 new cases of melanoma skin cancer and more than 7000 death of melanoma will occur in 2020 [3]. 

UVR can be especially hazardous to children as children and adolescents are generally considered to be more vulnerable to sunlight [2]. Children’s skin has a lower concentration of protective melanin [4,5] which allows UVR to reach deeper, resulting in more photodamage [4]. There has been an annual increase in melanoma of between 2 and 2.9% among children since 1970 [6]. 

Quantifying the distribution of UV radiation in a given landscape and mapping solar radiation on humans can help elucidate the relationship between UV exposure and children’s health and guide the design of sun protection programs. Current methods for estimating individual UVR exposure include field measurements using personal dosimeters, simulations based on the human body and solar position modeling, and satellite-based approaches. However, these methods have their limitations:

(1) Photosensitive personal dosimeters and polysulphone film UV dosimeter are instruments that are frequently used to quantify an individual’s UV exposure [7,8]. They are light, commercially available and can be mounted on body part to study anatomical distribution of UVR [9]. However, their measurements are strongly related to the specificities of local environmental conditions and behavioral variations [8,10,11]. Moreover, most dosimeters are mounted on an individual’s shoulders, wrist, or head and therefore measure UVR from one direction (mostly above), which has been shown to underestimate full-body UVR exposure [6,8]. Recently a UVR diary app was developed to estimate personal UVR exposure, but it needs further improvement, especially for physical activity data [12].
(2) Climatological approach using satellite data for the population UVR exposure level of national or regional scale can be used to demonstrate large area UVR condition. However, with a coarse resolution (e.g., 1.5 to 2 km), this approach can hardly be applied in site scale to reflect the human level UVR exposure and the interaction between the environment and the human body.
(3) Some three-dimensional computer graphics techniques are used to estimate UVR exposure ratios (ERs), while referring to the fraction of the ambient UVR received by the body area, for different body parts based on anatomical and geometric calculations. Computer modeling of ER for the whole body and specific body parts has a high resolution of vertices on human skin (e.g., 13,476 vertices for the SimUVEx model developed by Religi et al. [13]) and, compared to field measurements, performs with high accuracy in terms of predicting the ER of a human body in an open area. However, this model is not applicable to real sites with complex shade conditions such as those provided by structure or vegetation. Additionally, the frequently used manikin model is designed and calculated for adults. No evidence has been found regarding the difference or uniformity of UVR exposure between children and adults. Due to children’s vulnerability in UVR risk assessments, special attention must be paid to the estimation of children’s UVR exposure.

This study thus provides a new approach of integral calculation using six-directional (up, down, south, north, east, and west) field-measured UVR data and an estimated body exposure ratio (ER) to calculate both children’s and adults’ UVR exposure in a given landscape. Results are analyzed to (1) identify children’s and adults’ whole body ERs in different seasons in consideration of clothing conditions and solar positions, (2) construct a model to predict individual UVR exposure for children and adults, and (3) assess the model’s performance and limits. 

### Literature Review

Increasing epidemiological studies have supported the relationship between sunlight UVR exposure and enhancement of skin damage. Continuous exposure to sun or artificial UVR intentionally will increase the risk of getting skin cancer. However, the public awareness of the risk is not optimal, skin cancer rates continues to grow each year in all age groups, including in the younger population [2]. 

The variability of solar UV radiation at the Earth’s surface depends on the combined effect of several factors. For instance, atmospheric gases and aerosols, ozone column, cloud cover, solar zenith angle and distance from the sun (season and time), latitude, longitude and altitude, surface albedo, trees, and other objects in a given landscape [14]. An individual’s UV exposure is related to the direct, diffuse and reflected UVR irradiance, body posture and activity, duration of exposure in the open, skin type, and protective behavior [13,15]. 

UVR exposure during childhood is a critical period for the increase in skin cancer risk later in life [4,16]. An epidemiological study revealed that people who migrated from low UVR areas to high UVR areas at an older age had a lower risk of getting skin cancer compared to those who arrived at younger ages or as children [17,18]. Among US children, the incidence of sunburn is high: 29% to 83% over the entire summer season [19,20]. In 2010, approximately one third of US teens (14 to 17 years old) reported having had a sunburn during the previous 12 months [20]. Due to the differences in body characteristics of children, their UVR exposure calculations were separated from those of adults in this study.

Electronic or personal UV dosimeters have been tested and used in various studies for the measurement of personal UV exposure [10,15,21,22]. Wrists and shoulders were found to be valid sites on the body for mounting personal dosimeters in previous studies (e.g., Vanos et al. [15]). However, a measurement made by personal UV dosimeters facing upwards was found to underestimate full UVR exposure [15]. Due to direct, diffuse, and reflected UV irradiances, inclined surfaces hold a substantial amount of radiation. Measuring only horizontal surfaces is thus an inadequate indicator of environmental irradiance [23,24].

Moreover, personal UVR dosimeter measurements, such as photosensitive dosimeters and polysulphone film band dosimeters, are strongly related to an individual’s specific position and environmental shade conditions, are costly, and are prone to behavioral biases [8,13,21,22]. For example, 196 children in Stockholm, Sweden were asked to carry dosimeters and pedometers in different outdoor environments from May–June 2004 to test their daily UVR exposure ratio (ER) and UVR exposure. The range of ER in environments with trees and little vegetation were as wide as 4–60% [22]. Pagels et al. [21] conducted a similar experiment in Sweden among 2nd, 5th, and 8th graders to determine their daily UVR exposure. ER and sky view factors were found to be uncorrelated among the 8th graders but positive for the 2nd graders and negative for the 5th. Previous studies like these show that it is difficult to identify a consistent and quantitative relationship between individual UVR exposure and ER in a specific environment due to the complexity of the environment’s shade conditions and daily behaviors. 

Numerous efforts have been made to calculate solar irradiance in the directions of typically oriented surfaces of the human body using three-dimensional computer graphics techniques and calculations. For instance, a study by Pope and Godar [25] provided a geometric conversion factor to convert horizontal erythemal UV irradiance to a cylinder model to represent human body UVR exposure. However, this study didn’t consider human seasonal clothing condition, and wasn’t validated. Streicher et al. [26] produced a sun-tracing and irradiance algorithm to calculate UVR exposure of each anatomical area of human body. Hoeppe et al. [27] also developed a virtual three-dimensional measuring system, ASCARATIS, to calculate UVR exposure of human skin, which contained about 20,000 triangles of the human body. Vernez et al. [28] used a 3D numeric model SimUVEx to compute daily UVR dose and ER for various body sites and body postures. A recent modification for this model was later made by Religi et al. [13] using a manikin with two resolutions (high: 13,476 vertices; low: 837 vertices) for 45 anatomical zones. These sophisticated models have other useful applied areas, such as identifying the risk of skin cancer at some specific body part, although reflecting whole body UVR exposure in a real outdoor environment is difficult.

A human UVR exposure ratio (ER) refers to the ratio between the UVR amount received by a specific body site and the corresponding UVR amount received by a flat horizontal surface at ground level [29]. It has been used in previous studies to estimate individuals’ UVR exposure by considering the ambient UVR [10,30]. For example, Downs and Parisi [30] developed an approach to estimate ER of each body part for different latitudes under solar zenith angles ranges (0°–30°, 30°–50°, and 50°–80°), although this approach didn’t consider human body posture and activities. Weihs et al. [10] determined ER for four different outdoor activities by measuring 10 different positions of the body. However, the result from the study is hard to be applied to different location under different solar positions. Vernez et al. [29] developed a general regression model for predicting ER for different anatomical body parts and validated it by comparison with the SimUVEx model. However, the estimation or measurements of the ER in these studies were made in an open environment, which can’t be used for complicated environmental shade conditions.

To collect more relevant environmental information on UV exposure of the whole human body, a new model calculating individual UVR exposure has been developed based on the principle of UVR transmission in the environment [31,32] and its interaction with human skin. Due to differences in body surface area between children and adults, two versions of UV exposure models were separately calculated for this study. The accuracy of the models is presented through a comparison of the results from Vernez et al.’s [29] regression model using locally measured UVR data in College Station, Texas. This model was compared to the SimUVEx model and showed a high agreement (R^2^ = 0.988). As such, it can be used to accurately predict ER and UVR amount based on available data, e.g., global UV erythemal irradiance measured at ground surface stations or inferred from satellite information. 

## 2. Materials and Methods

UVR levels within the built environment are dependent on the three dimensional surface reflectance, diffusion, and reflection from the environment. In order to measure the full amount of UVR from the environment, a six-directional measurement of UVR was developed and calculated based on the principle of the solar radiation interaction between human body and the environment [31]. Human body coverage percentage by clothes of different seasons was considered at the same time. In order to validate this approach, another approach estimating children’s and adults’ UVR exposures was compared in this study, using the ambient global UVR irradiance multiplied by the body ER calculated from a model previously developed and extracted by Vernez et al. [29]. In this study, the ambient UVR means the erythemal weighted UVR amount measured by a sensor oriented horizontally. 

### 2.1. Ambient UVR Data and Six Directional UVR Data Collection in College Station, TX

SHADE erythemal weighted UVR radiometers (Shade^®^ model V1.00, YouV Labs Inc., New York, NY, USA) [33] were used for both ambient and six-directional UVR measurements. A SHADE radiometer is a validated, high-performance wearable UV radiometer for measuring a UVR exposure in minutes during outdoor exposure, tested as the most accurate and sensitive of devices [34] compared with other UV radiometers including Band (Microsoft) [35], June (Netatmo) [36], Sunfriend (Sfunfriend Corporation) [37], UV Watch (Dokato) [38], Sunsprite (Goodlux Technologies) [39], or the EPA UV Index mobile application [40]. The effective detect spectrum is 280–400 nm, with an erythemal weight based on ISO17166:1999/CIE. The sensor is paired with a custom developed mobile using Java for Android system. Data were expressed as UV Index (UVI), a unitless number defined as 40 times the erythemally weighted UV irradiance, expressed in Watts per meter square (W m^−2^) [41], with a collection interval of 1 s. The original calibration of the sensor was conducted by Banerjee et al. [34], using the research-grade X1-4 radiometer by Gigahertz-Optik as a reference (see details at [34]). In order to reduce between-sensor variability, a field calibration was conducted on a sunny day, 14 February 2020. Sensors were arranged on horizontal flat ground at the Texas A&M University (TAMU) Architecture Quad from 11 a.m. to 6 p.m. (Longitude 30.618, Latitude −96.337). A quadratic function with the intercept forced to 0 [15] was used for the sensors’ measurements to reduce between-sensors variability.

Ambient UVR data was measured using the SHADE sensor on the horizontal flat roof of Langford Abuilding at TAMU. Six other SHADE radiometers were mounted on a box facing six directions (up, down, north, south, east, and west) at the height of 1.5 m on a tripod on the roof of Langford A. Data was collected on a sunny day from 8:30 a.m. on 25 February to 9:00 a.m. on 26 February (Longitude 30.619, Latitude −96.338).

### 2.2. Exposure Ration Calculation for Children and Adults

Vernez et al.’s [29] equation and the visible percentage to the sky of specific body part (%) in different postures (seated, kneeling, standing straight arm up, standing straight arm down, standing bowing) was used in this study to predict ER for each body part (Table 1). The visible percentage took shading form other body parts and body orientations into account. For example, the 100% visible percentage meant that the body part is oriented upward, horizontal, and unshaded [29]. To calculate the whole-body ERs for children and adults separately, the percentage of surface area for each body to the total body surface area was based on O’Sullivan and Schimitz’s [42] Physical Rehabilitation 5th edition and Boniol et al.’s [43] results.

The whole-body ERs for a child and for an adult were calculated separately as shown below:

(1) $$ER_{bC}\;\mathrm{or}\;ER_{bA}=\overset{11}{\underset{i=1}{\sum }}ER_{P_{i}}\times SP_{part_{i}}$$

where $ER_{bC}$ (%) is the whole-body ER for a child, $ER_{bA}$ (%) is the whole-body ER for an adult, $ER_{P_{i}}$ (%) is the ER for a specific body part, and $SP_{part_{i}}$ (%) is the percentage of the surface area for a specific body part out of the total body surface area that is exposed to the environment. Seasonal clothing conditions were considered: in summer, $ER_{P}$s of belly, torso, and back were 0; in winter, $ER_{P}$s of arms, legs, and trunk were 0; in spring and autumn, $ER_{P}$s of limbs, trunk, and upper arm were 0.

### 2.3. Estimation of Child and Adult UVR Exposure by Ambient UVR Irradiance and ER

The estimation of child and adult UVR exposure by ambient UVR irradiance and ER were calculated as shown below:

(2) $$UVR_{ab}=UVR_{am}\times ER_{b}$$

where $UVR_{ab}$ is the UVR received for the whole body, $UVR_{am}$ is the ambient UVR irradiance and $ER_{b}$ is the whole-body ER for a child or an adult.

### 2.4. Estimation of Children’s and Adults’ UVR Exposure by Integral UVR Measurement

According to the principle of radiation transmission and the reaction between human skin and UVR, a six-directional integral calculation was based on Environmental Meteorology Guideline 3789 of German Engineering Society [32] and modified for the following equation:

(3) $$UVR_{int}=P_{c}\times \overset{6}{\underset{i=1}{\sum }}K_{i}F_{i}$$

$UVR_{int}$ = UVR exposure of the whole body by integral measurement

$P_{c}$ = seasonal clothing coefficient

$K_{i}$ = the UV radiation fluxes (i = 1–6)

$F_{i}$ = the angular factors between a person and the surrounding surfaces (i = 1–6)

Based on a cylindrical model [44],$\;F_{i}$ was calculated for children and adults separately. The top of the head, shoulders, and feet were considered to be areas facing up, while the trunk, arms, and legs were considered vertical surfaces. $P_{c}$ was the percentage of the surface covered by clothes. To be consistent, we use the same clothing conditions as in Equation (1): in summer, we assumed that belly, torso, and back will be covered by clothes ($P_{c\;}$ was 68% for children, 66% for adults); in winter, we assumed arms, legs, and trunk will be covered by clothes ($P_{c}$ was 18% for children, 14% for adults); in spring, we assumed legs, trunk, and upper arms will be covered ($P_{c}$ was 42% for children and 40% for adults).

### 2.5. Field Measurement in Schoolyards

To reveal the UVR environment in a schoolyard, the same suite of SHADE sensors was used to measure the ambient and six-directional UVR amount in Pebble Creek elementary school basketball court. The sensor measuring ambient UVR was oriented horizontally in the open yard, six other sensors were mounted on a box facing six directions at the height of 1.5 m on a tripod. The fraction of visible open sky (Sky View Fraction—SVF%) was determined by fisheye photography of the sky 1.5 m above the ground with the same pole position (North = upper photo position). The fraction of visible free sky in the north, south, east, and west directions of the spot (Side View Factor—SiVF) was also taken by the same method with the camera standing vertical facing the given direction. The camera used to take these photos was a Canon D6 (Canon Inc., Tokyo, Japan) and the lens was the Rokinon F3.5 (Elite Brands Inc., New York, NY, USA) fisheye lens.

## 3. Results

### 3.1. Anatomical Body Part ER for Children and Adults during Four Seasons in College Station, Texas

Daily maximum solar zenith angle affected $ER_{P}$ based on Vernez’s equation [29]. The local daily maximum cosine solar zenith angle (cSZA) calculation was based on the NOAA online solar position calculator [45] for a summer day (19 July 2019, maximum cSZA was 0.987), a winter day (19 January 2020, maximum cSZA was 0.641), a spring day (25 February 2020, maximum cSZA was 0.779), and an autumn day (19 October 2019, maximum cSZA was 0.763). $ER_{P}$s were calculated for each body part on different seasonal days (Table 2).

The highest $ER_{P}$ was on the shoulder (mean = 56.62%), which had the largest visible exposure part facing up. The lowest $ER_{P}$ was on the belly (mean = 35.29%) due to the shadowing of other body parts. $ER_{P}$ on the upper parts of arms and legs was higher than that of the lower parts. $ER_{P}$ for the anterior torso (bosom and belly, 38.26%) was lower than that of the posterior torso (upper back and lower back, 45.80%), indicating that there were more shadows on the front part of the body.

$ER_{P}$ of each body part on 19 January 2020 (from 35.57% to 56.9%) was the highest among the four days due to a lower solar zenith angle and was the lowest on 19 July 2019 (from 33.51% to 54.83%) due to a higher solar zenith angle.

Table 3 demonstrates the seasonal results for $ER_{b}$ children and $ER_{b}$ adults. With the same clothing coverage, children’s and adults’ $ER_{b}$ were similar across all four seasons. With the bosom, belly, and back considered covered by clothes (ER = 0), summer $ER_{b}$ was the highest with 28.73% for children and 28.27% for adults, relatively. The whole-body ER values for children and adults were later used to calculate individual UVR exposure.

### 3.2. Comparison of Two Approaches for Measuring UVR Exposure Based on Field Measurement Data on 25 February 2020

**Figure 1:**
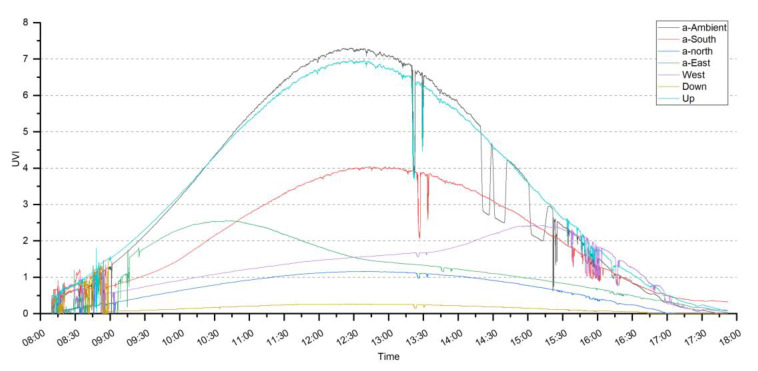
Six-directional ultraviolet radiation (UVR) daily data.

Figure 1 shows the six directional UVR data collected from 8:30 a.m. to 5:30 p.m. on 25 February 2020 in College Station, Texas. Table 4 shows descriptive statistics of the UVR values. The daily minimum UVR values for the six-directional UVR data are 0. Daily maximum ambient UVR was 7.31 UVI (182.75 mW/m^2^) at 12:33 p.m., similar to that of the tripod-mounted sensor facing up (6.96 UVI, 174 mW/m^2^). More than half of the UVR came from the southern sky (53.9% of the maximum ambient amount and 58.7% of the mean ambient amount), especially at noontime when the ambient UVR reached its peak value. UVR from the eastern sky reached its peak value in the morning at 10:33 a.m. (3.14 UVI, 78.5 mW/m^2^), while the peak value of the western sky was reached in the afternoon at 3:30 p.m. (2.28 UVI, 57 mW/m^2^). At 9:00 a.m. and 4:00 p.m., the amount of UVR from the eastern and western skies were almost the same amount from overhead. The average ratio of each direction to the ambient UVR was 1.01 for up, 0.56 for south, 0.41 for east, 0.30 for west, 0.21 for north, and 0.07 for down.

**Figure 2:**
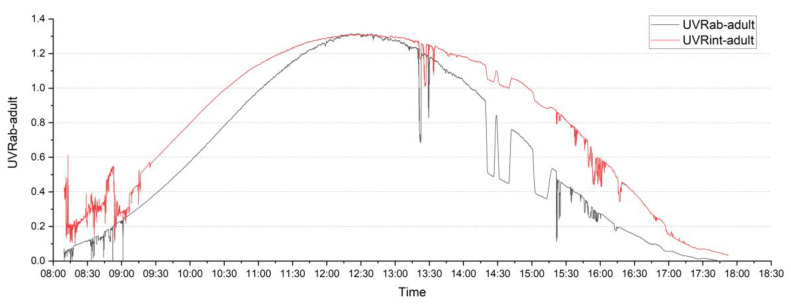
Adults’ UVRab and UVRint on 25 February 2020.

The relationship between the two approaches of estimating individual UVR for adults was compared using estimated ER and the ambient irradiance UVR data ($UVR_{ab}$), as well as integral calculation of UVR data from six directions ($UVR_{int}$) (Figure 2). The cSZA on 25 February 2020 was calculated according to the NOAA online solar position calculator every six minutes [45]. The maximum $UVR_{ab}$ for an adult was 1.32 UVI (33 mW/m^2^), and $UVR_{int}$ was 1.31 UVI (32.75 mW/m^2^). The daytime, eight-hour (8:30 a.m. to 5:30 p.m.) maximum erythemal dose was 2.3 MED (555 J/m^2^) based on $UVR_{ab}$ and 3.5 MED (707 J/m^2^) based on the $UVR_{int}$ results for adults. Daily cumulative UVR data based on $UVR_{ab}$ was higher than $UVR_{int}$, but not significant at *p* < 0.01. The shape of $UVR_{ab}$ was steeper and more like the environmental UVR curve in Figure 1. The multi-directional measurements seemed to have a ‘smoothing’ effect on the curve. In morning and afternoon times, values of $UVR_{int}$ were higher than $UVR_{ab}$ due to the influence of side directional UVR on the $UVR_{int}$ value.

When looking at children’s UVR exposure using the two approaches of ambient UVR times—ER ($UVR_{ab}$), and six-directional measurements ($UVR_{int}$)—the maximum $UVR_{ab}$ was 1.31 UVI (32.75 mW/m^2^), and $UVR_{int}$ was 1.35 UVI (33.75 mW/m^2^). There were no significant differences between children’s and adults’ results for both $UVR_{ab}$ and $UVR_{int}$. The daytime eight hours maximum erythemal dose was 2.3 MED (550 J/m^2^) based on $UVR_{ab}$ and 3.6 MED (732 J/m^2^) based on the $UVR_{int}$ approach for children. No significant difference was found between $UVR_{ab}$ and $UVR_{int}$ for children.

**Figure 3:**
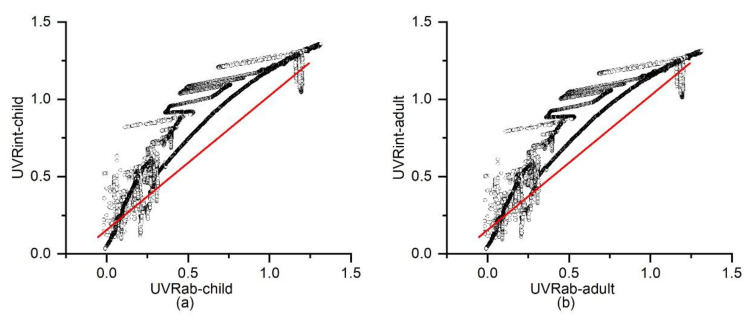
(**a**) Correlation between UVRab and UVRint for children, (**b**) UVRab and UVRint for adults. The red line illustrates the linear fitted line.

Figure 3 shows the correlation between the individual UVR exposure results from $UVR_{ab}$ and $UVR_{int}$ for children and adults. The overall agreement between the two approaches was high in both child and adult models. The Pearson correlation coefficient of the children’s models was 0.95 at *p* < 0.01, the same as the adults’ model, which was calculated by IBM SPSS 24. Linear regression was applied to demonstrate the relationship between the two models for both children and adults (the red line shows the fitted line). R-square was more than 90% for both conditions. Mean square error (MSE) was used to demonstrate the differences between the two ER models [13,29]. The MSE was 6.0% for the children’s model and 5.1% for the adults’ model, indicating high agreement between the two approaches in calculating individual UVR exposure under an open sky environment.

### 3.3. Environmental UVR Amount of a Basketball Court

Table 5 shows the UVR amount from five directions under the central of a canopy of a basketball court in College Station, TX. A considerable amount of UVR comes from the southern, eastern, and western skies, indicating that, in landscape scenarios, UVR still comes from other directions, even if the sky has been covered by canopies. The UVR coming from the southern sky was four times higher than from above, as the sky view factor was 0 (totally covered by the constructed canopy). At the same time, the south direction was still open to the sky, allowing UVR to reach to the human body under the canopy. In this case, using a dosimeter mounted on the shoulder or wrist underestimates the actual UVR exposure for a human body.

## 4. Discussion

This study used a previously validated regression model to calculate children’s and adults’ ER for 11 body parts on four days of summer (19 July 2019), winter (19 January 2020) spring (25 February 2020), and autumn (19 October 2019) in College Station, TX. The whole-body ER was estimated for children and adults considering various conditions of body surface area coverage. Over the four days, ER of the shoulder was highest and ER of the belly was lowest. On the summer day, children’s and adults’ ERs were as high as 28%; on the winter day, the ER was 6–8% for both. Overall, there were no significant differences between children’s and adults’ ER on the four days.

Based on six directional UVR measurements facing up, down, south, east, west, and north in the daytime, a considerable amount of UVR came from the southern, eastern, and western skies. When compared to ambient UVR, the UVR from the south was higher than 55% and the UVR from the east and west were 30–40%. This amount of UVR has always been ignored by previous studies.

Based on the principle of UVR transmission, an integral calculation of the six directional UVR data’s interaction with human skin was developed in this study to estimate individual UVR exposure in an open area. Seasonal clothing conditions were considered via the percentage of skin covered by clothes. This approach was compared with the extant method of using ambient UVR multiplied by body ER. The two approaches showed high agreement with MSE (around 6%), with R-square of more than 90% for both child and adult models. Given this, the technique of using six directional sensors was found to be an accurate, high performing approach for calculating individual UVR exposure in an open space.

Previous studies that have estimated whole-body ER or anatomical body parts have been conducted in open areas with a full sky view. They are thus not applicable in real landscapes with shade structures and vegetation. Due to the complexity of the physical environment and individual behavior bias, ER results have been shown to vary greatly from field measurements [8]. This indicates the complexity of using field measurements and computer modeling estimation to identify quantitative relationships between ER, daily activity, and the physical environment. In this study, we used both field measurements to get the real UVR irradiance of the environment and skin coverage by clothing to obtain accurate estimations of individual UVR exposure in a real landscape. In addition, children’s and adults’ conditions were calculated separately. Overall, we found this approach to be a temporally and commercially practical approach to estimating and predicting individual UVR exposure in a landscape for children and adults.

### Limitations

Body posture and activity have been shown by previous studies to have critical effects on body ER. Due to limited data on children’s ER for specific physical activities, we applied the average ER for five postures in this study. However, the regression and computing models were designed for adults. Thus, there are still no validated ER value for children in open areas. Moreover, children are usually engaged in activities that are more intensive than sitting, standing, or bowing when playing outside. Based on previous studies, more intensive activity leads to less ER. The results of this study may thus overestimate total UVR exposure. Furthermore, only one day of data in February 2020 was used to test the results from the approach in this study. Future studies should measure multiple days from summer, winter, and autumn to verify this model in a variety of climates and seasons.

## 5. Conclusions

This study developed a landscape-based approach, based on measurements from six directional UVR radiometers, to estimate individual UVR exposure. It is an intermediate approach between personal dosimetry and the use of ambient irradiance data. The use of several stationary radiometers allowed us to better take into account the sun orientation effects and local landscape features (e.g., ambient shading, albedo) without the need for tedious individual measurement. It makes it possible to assess, with reasonable means of investigation, the exposure of a group of individuals confronted with similar local conditions. Results obtained by this technique showed high agreement for both children’s and adults’ exposure data with the computation of individual exposure through the use of ER and ambient UVR data.

## Figures and Tables

**Table 1:** Visible percentage and percentage of surface area for body parts.

	Visible Percentage (%)	Percentage of Body Part Surface Area for an Adult (%)	Percentage of Body Surface Area for a Child * (%)
Face	38.62	3.9	6
Skull	61.96		
Forearm (external)	56.68	5.9	5.7
Upper arm (external)	57	9.6	8.6
Neck back	71.4	2	2.7
Top of shoulders	53.16	12.8	12.2
Belly	43.66	2.9	2.8
Upper back	53.16	13.9	13.1
Low back	58.68		
Hand (back)	54.78	4.7	4.7
Shoulder	64.82	1.9	1.9
Upper Leg (front)	51.24	18.3	17.6
Lower leg (back)	49.24	11.2	11.7

* Values are the average value for an 8-year-old boy and girl from Boniol et al. (2008).

**Table 2:** Exposure Ration (ER) for 11 body parts in four seasonal days.

	${\mathrm{ER}}_{p}\;\mathrm{Summer}$ (%)	${\mathrm{ER}}_{p}\;\mathrm{Winter}\;$ (%)	${\mathrm{ER}}_{p}\;\mathrm{Spring}$ (%)	${\mathrm{ER}}_{p}\;\mathrm{Autumn}$ (%)	$\mathrm{Mean}\;{\mathrm{ER}}_{p}\;\mathrm{for}\;\mathrm{Each}\;\mathrm{Body}\;\mathrm{Part}$ (%)
Head	40.14	43.31	42.04	42.19	41.92
Forearm	46.57	49.75	48.49	48.64	48.36
Upper arm	46.89	50.08	48.81	48.96	48.68
Bosom	43.02	46.20	44.93	45.08	44.80
Belly	33.51	36.69	35.42	35.57	35.29
Upper back	43.02	46.20	44.93	45.08	44.80
Hand	44.65	47.83	46.57	46.71	46.44
Shoulder	54.83	58.02	56.76	56.90	56.62
Upper Leg	41.08	44.27	43.00	43.15	42.8
Lower leg	39.08	42.26	40.99	41.14	40.86
Low back	48.59	51.78	50.51	50.66	50.38
Average	43.76	46.94	45.68	45.83	

**Table 3:** Whole-body ER for children and adults in different seasons.

	$ER_{bC}$ (%)	$ER_{bA}$ (%)
Summer	28.73	28.27
Winter	8.04	6.75
Spring/Autumn	16.98	16.68

**Table 4:** Descriptive Statistic of six directional UVR values.

	Maximum (Proportion to Ambient Value%)	Mean (Proportion to Ambient Value%)	Std. Deviation
Ambient	7.31	3.41	2.58
Up	6.96 (95.2%)	3.49 (102.3%)	2.37
South	3.94 (53.9%)	2.00 (58.7%)	1.31
north	1.29 (17.6%)	0.46 (13.5%)	0.41
East	3.14 (43.0%)	1.05 (30.8%)	0.79
West	2.28 (31.2%)	1.11 (32.6%)	0.66
Down	1.14 (15.6%)	0.13(3.8%)	0.20

**Table 5:** Pebble Creek Elementary School (College Station, TX) basketball field UVR and Sky/Street view factor measurement in 5 directions.

	Up	South	North	East	West
	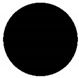	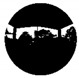	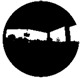	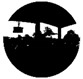	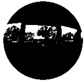
SVF/SiVF	0%	9.10%	13.53%	14.50%	8.63%
UVI	0.15	0.63	0.57	0.54	0.75
